# Mechanisms of Chemopreventive and Therapeutic Proprieties of Ginger Extracts in Cancer

**DOI:** 10.3390/ijms22126599

**Published:** 2021-06-20

**Authors:** Mariia Zadorozhna, Domenica Mangieri

**Affiliations:** Department of Medical and Surgical Sciences, University of Foggia, Via Pinto 1, 71122 Foggia, Italy; mariia.zadorozhna@unifg.it

**Keywords:** ginger extracts, chemoprevention, chemotherapy, natural compounds

## Abstract

Ginger (Zingiber officinale Roscoe, family: Zingiberaceae), originating in South-East Asia, is one of the most used spices and condiments for foods and beverages. It is also used in traditional medicine for many human disorders including fever, gastrointestinal complications, arthritis, rheumatism, hypertension, and various infectious diseases due to its anti-inflammatory, antioxidant, antimicrobial, and antiemetic properties. Intriguingly, many recent studies evidenced the potent chemopreventive characteristics of ginger extracts against different types of cancer. The aim of this work is to review the literature related to the use of ginger extracts as a chemotherapeutic agent and to structure the cellular and molecular mechanisms through which ginger acts in different cancer types. Data summarized from experiments (in vitro or in vivo) and clinical studies, evidenced in this review, show that ginger derivatives perpetrate its anti-tumor action through important mediators, involved in crucial cell processes, such as cell cycle arrest, induction of cancer cell death, misbalance of redox homeostasis, inhibition of cell proliferation, angiogenesis, migration, and dissemination of cancer cells.

## 1. Introduction

Cancer, being a multifactorial disease, is the second biggest cause of death in the world despite a great development of different types of its treatment [1]. Therapeutic options against cancer include surgical procedure, radiation therapy, chemotherapy as well as target and gene therapy [2]. Since the currently available treatment options are often accompanied by severe toxicity and side effects, consequently, researchers are consistently searching for new therapeutic solutions [3].

In this regard, 50% of approved cancer therapeutic agents are derived from natural products and, secondarily, medicinal plants metabolites have demonstrated a great perspective as a source of anticancer and chemopreventive compounds [4]. Composts isolated from edible plants have the advantage of low toxicity profiles and can simultaneously target multiple signaling pathways [5]. Therefore, dietary natural products can provide novel and fascinating preventive/therapeutic options for different kinds of neoplasia.

**Figure 1:**
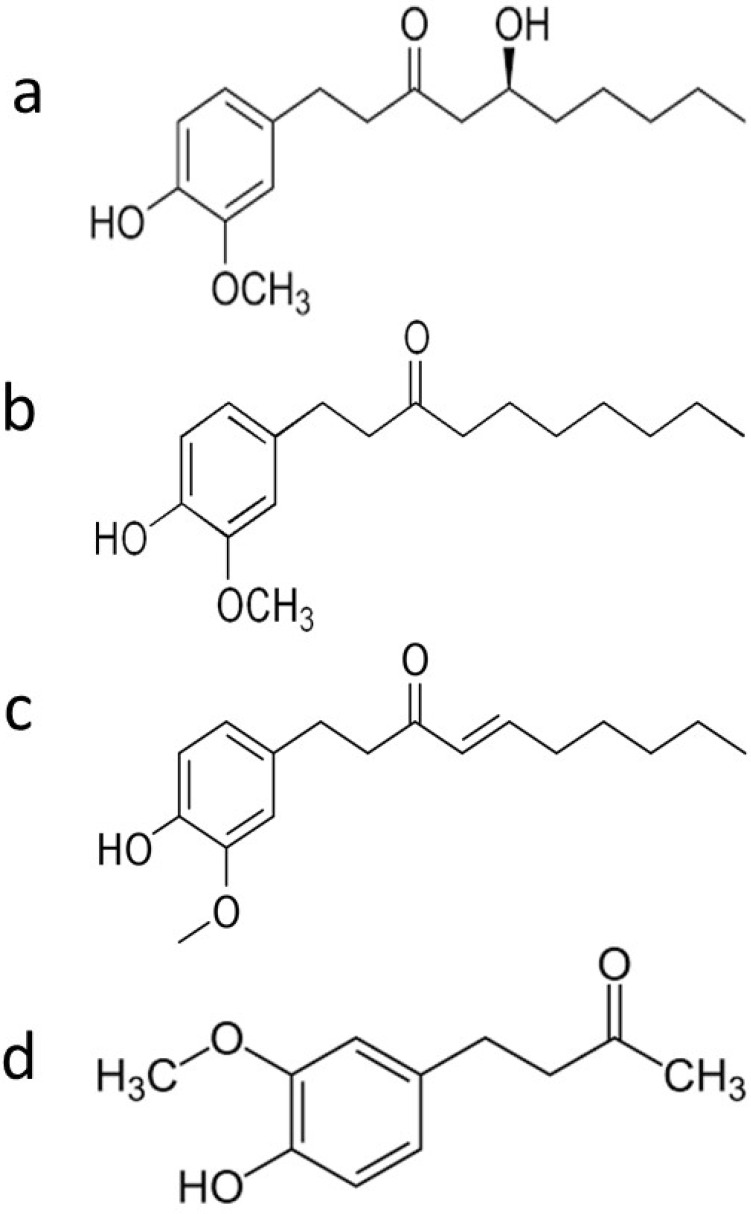
Chemical structure of main phenolic compounds of ginger: (**a**) 6-gingerol (1-[4’-hydroxy-3’-methoxyphenyl]-5-hydroxy-3-decanone), (**b**) 6-paradol, (**c**) 6-shogaol, and (**d**) zingerone.

Ginger is known for having more than 60 active compounds, broadly divided into volatile and nonvolatile compounds [6]. Volatile components include hydrocarbons, meanwhile rhizome from ginger contains nonvolatile pungent phenolic compounds like 6-gingerol, 6-shagol, 6-paradol, and zingerone [7] (Figure 1, Table 1).

**Figure 2:**
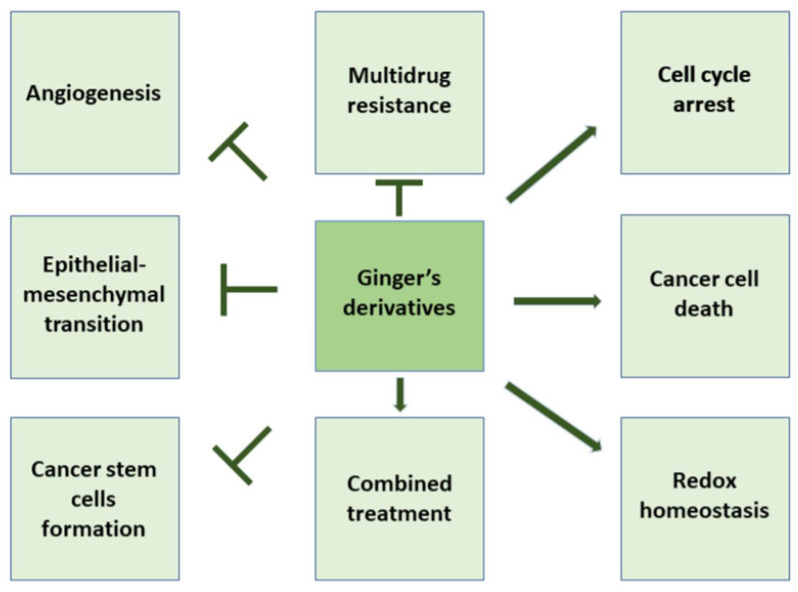
Schematic anti-cancer action of ginger and its phenolic derivatives. This natural compound reduces cancer cell proliferation, arrests cell cycle, causes an imbalance in cellular redox homeostasis, and induces cell death. Additionally, ginger derivatives inhibit angiogenesis, EMT, and CSCs. Furthermore, they decrease multidrug resistance and enhance chemopreventive effects.

**Figure 3:**
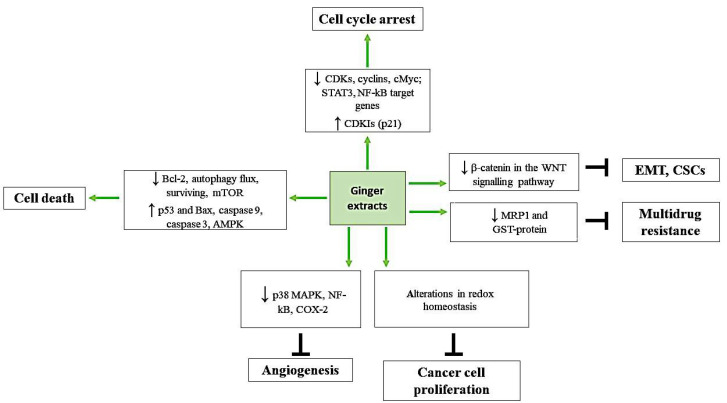
A schematic diagram showing main molecular targets of ginger derivatives. The natural compound participates in the cell cycle arrest by inhibiting the expression of cyclins, CDKs, and levels of STAT3, NF-kB target genes, and by activation of cell cycle check points and increased expression of p21. Moreover, by increasing Bax/Bcl-2 ratio outburst of cytochrome C, activating AMPK, and by decreasing autophagy flux and survivin expression, ginger derivatives provoke cancer cell death. They participate in the alteration of redox homeostasis, which stops cancer cell proliferation. By blocking activation of p38 MAP kinase (p38 MAPK) and NF-kB, ginger derivatives inhibit COX-2 expression and as a result block angiogenesis. Ginger extracts decrease β-catenin in the WNT signaling pathway, which leads to the inhibition of gene transcription, involved in EMT and CSCs, and, additionally, downregulate MRP1 and GST-protein expression, involved in multidrug resistance.

These exact compounds have been studied for their anti-bacterial, antioxidant, and anti-inflammatory properties [19]. Ginger phenolic compounds especially have also shown anti-tumor properties [9,20,21] (Figure 2). In this review, we will more deeply discuss the chemopreventive molecular mechanisms of ginger derivatives including arrest of the cell cycle, induction of cancer cell death, misbalancing of redox homeostasis, inhibition of cell proliferation, angiogenesis, migration, and dissemination of cancer cells in different cancer types (Table 2, Figure 3).

## 2. Ginger Derivatives and Cell Cycle Arrest

Cell cycle is critical to maintaining cell proliferation and tissue integrity; therefore, it is thoroughly controlled in defined checkpoints by specific proteins and kinases that include cyclins, cyclin-dependent kinases (CDKs), and CDK inhibitors (CKIs) [30]. Deregulation of cell cycle and/or its arrest are often responsible for cancer onset and progression [30]. Experimental studies demonstrated that some ginger derivates were able to modulate cell cycle progression as a part of their chemopreventive mechanism [40] (Figure 2) For example, in recent work regarding the treatment of breast cancer cells (MCF-7 and MDA-MB-231) with 6-shogaol, the arrest of the cell cycle in G2/M phase was reported in both monolayer and cancer-stem cell-like spheroids, and in the last experimental setting, 6-shogaol also interfered with the stem cell self-renewal pathway [13]. In another study, ginger extract arrested cell cycle at G0/G1 and G2/M phases in the HT 29 colon cancer cell line at a low concentration (455 µg/mL), whereas in the HCT116 colon cancer cell line, the same effect was reached at a higher concentration (496 µg/mL) due to the inhibitory effect on CDKs [41]. Furthermore, in the HT29 and HCT116 colon cancer cell lines, ginger extract treatment caused a significant reduction in cyclin D1 gene expression coupled with down-regulation of mTOR and Wnt/β-catenin pathways and consequent cell cycle arrest in the G0/G1 phase [41,42]. In addition, it was reported that human colon cancer cells (HCT116) treated with R. stricta (CAERS) and crude flavonoid extracts from Z. officinale (CFEZO) acted synergistically in cycle progression by inhibiting cMyc and the Cdk4/cyclin complex while upregulating p21 expression, a transcriptional target of p53 [43]. Saha and co-workers observed that cancer prostate cell lines (human PC3; DU145; LNCaP and murine HMVP2) treated with 6-shogaol, showed decreased levels of several signal transducer and activator of transcription 3 (STAT3) and NF-κB-regulated target genes including cyclin D1 [14]. In addition, zerumbone (a sesquiterpene derived from the ginger plant Zingiber zerumbet) caused the arrest of cell cycle in the G1 phase in human prostate cancer cell line (DU-145) culture [14]. Furthermore, zerumbone caused a cell cycle arrest at G2/M (in a dose-dependent manner) by inhibiting PI3K/AKT/mTOR and STAT3 in hepatocellular carcinoma (human HepG2, Hep3B, Sk-Hep-1, SNU-182, SNU-449, Huh-7, and MHCC-LM3 cell lines, and murine Hepa1 cell line) [25]. The same compound impeded shunting of glucose-6-phosphate through the pentose phosphate pathway, thereby forcing tumor cells to undergo a cell cycle arrest. In human gastric adenocarcinoma (AGS), the 6-gingerol treatment showed a notable increase in the percentage of cells in the G2/M phase, accompanied by a resultant decrease of cells in the SubG0 phase [8].

Another study showed that zerumbone (ZER) administration significantly retarded the growth of orthotopic MDA-MB-231 xenografts in severe combined immune-deficient (SCID) mice [44]. The antitumor effect of ZER in vivo was accompanied by reduced cell proliferation as evidenced by Ki-67 (proliferation marker) expression and increased apoptosis. Additionally, ZER administration was well-tolerated by the mice and did not cause weight loss or any other side effects.

Based on the above-mentioned observations, it seems evident that ginger derivatives interfere with the proliferation and cell cycle of cancer cells by arresting cell cycle in G0/G1 or G2/M phases, by significantly reducing the cyclin D1 gene expression, by upregulating p21 expression, and by inhibiting PI3K/AKT/mTOR and STAT3.

## 3. Ginger and Cellular Death

Apoptosis, or programmed cell death, evolved as a rapid and irreversible process to efficiently eliminate dysfunctional cells [31]. Apoptosis is usually executed in two ways: Mitochondria-mediated intrinsic pathway and death receptor-mediated extrinsic route. In this process are involved cysteine-aspartate proteases (caspases) and the Bcl-2 family proteins (e.g., Bax, Bcl-2) [29,32]. Moreover, it is well known that in pathological conditions such as cancer, alterations/mutations in the p53 gene are one of the main causes of apoptosis changes [33]. As matter of fact, studies effected by Pashaei-Asl et al. showed that the treatment of ovarian cancer cell line SKOV-3 with ginger extract for 48 h caused the decrease of Bcl-2 gene expression, and the subsequent p53-induced apoptosis [45]. In another study, depolarization of the mitochondrial membrane and its potential subsequent deterioration (ΔΨm) were observed after 6-gingerol administration within the human gastric adenocarcinoma cell line (AGS) [8]. Interestingly, disruption of the mitochondrial permeability through the transition pore with decrease in ΔΨm is one of the pivotal events in cell response to apoptotic stimuli [34]. Mitochondria-mediated apoptosis in 6-gingerol-treated AGS cells was followed by cytochrome c release, elevation in the Bax/Bcl-2 ratio, and activation of caspases-3 and -9 [8]. Aqueous extract of ginger (GAE) induced cellular apoptosis and disrupted cellular interphase microtubules also within human non-small lung epithelium cancer (NSCLC) A549 cell lines, by increasing the Bax/Bcl-2 ratio and activating the mitochondrial death cascade [46]. In addition, 6-shogaol induced apoptosis in hepatocarcinoma cell lines (Hep-2) by loss of cell viability, enhanced ROS production, lipid peroxidation resulted in altered mitochondrial membrane potential, and increased DNA damage [15]. In particular, the prooxidant role of 6-shogaol seemed to inhibit Bcl-2 expression accompanied by an up-regulation of Bax, cytochrome c, released by altered mitochondria, and caspases-3 and -9 activation [15]. Similarly, β-Elemene, another extract from the ginger plant, triggered apoptosis in NSCLC through the cytochrome-c mitochondrial release, mediating the intrinsic apoptotic pathway [47]. Furthermore, torch ginger (*Etlingera elatior*, EE) induced caspase-independent cell death in mouse B16 melanoma cells through the inhibition of the ERK1/2, p38, and Akt signaling pathway [37]. In fact, it is well-known that the PI3K/Akt, ERK1/2, and p38 MAPK signaling pathways are crucial in the context of DNA-damaging drug-induced apoptosis [38]. Terpenoids present in the Steam Distilled Extract of Ginger (SDGE) induced apoptosis in endometrial cancer cells (ECC-1 and Ishikawa cell lines) at IC50 of 1.25 µg/mL by increasing the expression of p53 and Bax and simultaneously decreasing the expression of Bcl-2 by 90% [48].

Survivin is a member of the inhibitor of apoptosis (IAP) family and results in being up-regulated in different human cancers [35]. Interestingly, over-expression of this protein is associated with inhibition of apoptosis, resistance to chemotherapy, and a higher aggressiveness of tumors [49]. In this context, recent study showed that 6-shogaol, at 20 μM and 40 μM, provoked downregulation of survivin in head and neck squamous cell carcinoma (HNSCC) cell lines and consequently a significant increase in apoptotic death [16].

A very important fact is that there are some results of in vivo experiments confirming the positive role of ginger derivatives in apoptosis induction within cancer. In this setting, oral squamous cell carcinoma induced by painting with 0.5% 7,12-dimethylbenz[a]anthracene-induced (DMBA-induced) in hamster buccal pouch (HBP) (male golden Syrian hamsters) evidenced over-expression of the mutant form of p53 and Bcl-2 coupled with decreased expression of wild type p53 and Bax [17,50]. Oral treatment with 6-shogaol (at 20 mg/kg of body weight) showed significantly decreased tumor volume and tumor burden, restored wild-type p53 function, and activation of apoptotic stimuli [17].

Methanolic extract of *Zingiber officinale* rhizome (ZOME) induced morphological changes such as cell shrinkage and nuclear condensation demonstrating apoptotic properties of ZOME within cervical cancer HeLa and breast cancer MDA-MB-231 cell lines 1. Moreover, apoptosis of these cancer cell lines was gradually raised with an increasing order of concentration of extract, which revealed dose-dependent apoptosis [51].

Autophagy is a self-destructive process important for balancing sources of energy in the embryo development and as a response to several triggers of cellular stress (e.g., deprivation of growth factors/nutrients, inhibition of proteasome, inhibition of receptor tyrosine kinases/Akt/mammalian target of rapamycin (mTOR) signaling, and unbalance of ROS homeostasis) and is characterized by a cascade of events including degradation of cytoplasmic proteins or entire organelles [36,52,53]. Interestingly, the correlation between autophagy and cancer is controversial; in this context, it seems that autophagy could act as a tumor suppressor, provoking a programmed cell death of type II [22] and/or as tumor activator, directly affecting the cell–matrix focal adhesions (FAs), essential for efficient migration and invasion of cells [39].

Interestingly, co-treatment with gingerol and TRAIL of TRAIL-resistant A549 adenocarcinoma cells increased the LC3-II and p62 levels, that attested the inhibition of autophagy [54]. The gingerol treatment strongly enhanced apoptosis in TRAIL-resistant A549 cells, which was confirmed by the intracellular apoptosis indicator cleaved caspase-3. The results of this study suggested that gingerol sensitized TRAIL-induced apoptosis in A549 lung adenocarcinoma cells by inhibiting autophagy flux.

The semi-synthetic analogue SSi6, generated after chemical modification of the 6-gingerol molecule, using the acetone-2,4- dinitrophenylhydrazone (2,4-DNPH) reagent, enhanced selective cytotoxic effects on MDA-MB-231 (Triple negative breast cancer, (TNBC)) cells [55]. Remarkably, unlike the original 6-gingerol molecule, SSi6 enabled autophagy followed by caspase-independent apoptosis in tumor cells. A time-dependent association between SSi6-induced oxidative stress, autophagy, and apoptosis was reported. Initial SSi6-induced ROS accumulation (1 h) led to autophagy activation (2–6 h), which was followed by caspase-independent apoptosis (14 h) in TNBC cells. Additionally, the data showed that SSi6 induction of ROS accumulation played a key role in the promotion of autophagy and apoptosis [55]. Another experiment showed that breast cancer cells MCF-7 and MDA-MB-231 after 6-shogaol treatment underwent cell death, exploiting the autophagy route proved by cytoplasmic vacuole formation as well as the recruitment and cleavage of the microtubule-associated protein LC3 [13].

Another recent study showed that 6-Gingerol treatment in the human lung cancer cell line (A549) and in A549 tumor xenografts could increase the number of autophagosomes, ROS, and iron concentration, decrease the survival and proliferation rate of A549 cells, and significantly decrease tumor volume and weight [56]. Interestingly, 6-Gingerol treatment significantly suppressed USP14 expression, indicating that 6-Gingerol promoted autophagy effected by inhibition of USP14-Beclin 1. Remarkably, daily oral feeding of 100 mg/kg body weight of ginger extract (GE) inhibited growth and progression of PC-3 (prostate cancer) xenografts by approximately 56% in nude mice, as shown by measurements of tumor volume [57]. Tumor tissue from GE-treated mice showed reduced proliferation index and widespread apoptosis, as determined by immunoblotting and immunohistochemical methods, compared with controls.

Also, recent research demonstrated that the human pancreatic cancer (Panc-1) cell line treated with the extract of *Syussai ginger* (SSHE) revealed several features that were not observed in classical-type autophagy, including nuclear shrinkage, focal membrane rupture, electron dense mitochondria, empty vacuoles, and focal perinuclear swelling [58]. It appeared that these morphological features coincided well with the recently discovered form of cell death, “autosis”, which is a Na^+^ and K^+^ -ATPase-regulated form of cell death [23]. SSHE markedly increased the LC3-II/LC3-I ratio, decreased SQSTM1/p62 protein, and enhanced vacuolization of the cytoplasm in Panc-1 cells. So, SSHE inhibited cell proliferation and subsequently induced the autotic death of pancreatic cancer Panc-1 cells.

To sum up, the chemopreventive effect of ginger derivatives may be expressed by its ability to enhance some types of cellular death in cancer, like apoptosis, autophagy, and autosis by elevating Bax/Bcl-2 ratio, releasing cytochrome c, activating caspases-3 and -9, and downregulating the survivin.

## 4. Ginger, Its Constituents, and ROS Balance

Reactive oxygen species (ROS) are a group of highly reactive molecules generated through a variety of sources (mitochondria, NADPH oxidases (Nox), xanthine oxidase (XO), and uncoupled endothelial nitric oxide synthase (eNOS), lipoxygenase, cyclooxygenase, and CYP-P450s enzymes) [59,60,61]. Elevated ROS rates have been detected in almost all cancers, where they promote many aspects of tumor development and progression. However, tumor cells also express increased levels of antioxidant proteins to detox from ROS, suggesting that a delicate balance of intracellular ROS levels is required for cancer cell function [62].

A challenge for novel therapeutic strategies will be to direct the ROS signaling towards ROS-induced apoptotic route. In this scenario, recent studies showed that ZOME scavenged the ROS actions, in a dose-dependent manner, both in human cervical cancer (HeLa) cells and in breast cancer (MDA-MB-231) cells [51]. Another study reported that after treatment of AGS cells with 6-gingerol, an increase in the level of reactive ROS led to a decrease in mitochondrial membrane potential and consequent induction of apoptosis [8]. Also, the incubation of DU-145 prostate carcinoma cells with zerumbone led to a reduction of cell viability, in a dose- and time-dependent manner, by increasing the ROS production [63]. Additionally, Akimoto and co-workers demonstrated that the extract of Syussai ginger (SSHE) had a strong inhibitory effect on cell growth as well as pro-apoptotic activity in pancreatic cancer in vitro [58]. In particular, the authors showed that ROS production was suppressed in SSHE-treated Panc-1 cells at early stages that might be due to the antioxidant properties of the ginger extract [64]. However, prolonged treatment of cells with SSHE caused a marked increase in ROS production, which induced autotic cell death. The extract was also effective under hypoxic conditions, which inevitably develop in all solid tumors to varying degrees and influence the resistance of tumor cells to radiotherapy and conventional chemotherapy [64]. Recently, Kathiresanet et al. discovered that 6-shogaol (20 mg/kg body weight) had a potent anticancer activity against DMBA-induced oral carcinogenesis in the HBP model by restoring antioxidant levels, thereby preventing lipid peroxidation. In addition, 6-shogaol was also able to inhibit phase I enzymes (Cyt-p450 and Cyt-b5) and increase phase II enzymes (GST, GR, and GSH) that enhanced the detoxification, thereby preventing the carcinogenesis [17].

Another interesting clinical study had the principal objective to examine the antioxidant activity of ginger extract orally administered as a daily supplement in newly diagnosed solid tumor patients receiving moderate-to-high emetogenic potential chemotherapy [65]. All participants were women, of whom 39 patients (91%) were diagnosed with breast cancer who received anthracycline-based regimen, 24 patients (56%) were diagnosed with stage II, and 13 patients (30%) diagnosed with stage III. In all, 90% of patients had a good performance status (ECOG = 0). A daily supplement of ginger extract, started 3 days prior to chemotherapy, showed significantly elevated antioxidant activity and reduced oxidative marker levels in patients who receive moderate-to-high emetogenic potential chemotherapy compared to a placebo [65]. In subsequent cycles of chemotherapy, patients seemed to have significantly elevated oxidative defense status based on their higher blood levels of Cu-Zn superoxide dismutase (CuZn-SOD), catalase (CAT), glutathione (GSH/GSSG), and GPx couplet with significantly reduced levels of malondialdehyde (MDA) and NO_2_-/NO_3_- after continuously receiving ginger extract. This effect was not observed in patients who received placebos. Furthermore, patients taking ginger extract continuously were inclined to increase antioxidant enzyme blood levels and decrease oxidative stress blood level [65].

In summary, ginger derivatives could play an important role in maintaining redox homeostasis: In some cases, by decreasing the quantity of ROS-induced tumor-promoting events, and in other cases, in contrast, by increasing oxidative stress and provoking cell death.

## 5. Ginger and Angiogenesis

Angiogenesis, the formation of new blood vessels from pre-existing endothelium, depends on complex cellular activities, such as extracellular matrix degradation, proliferation and migration of endothelial cells, and morphological differentiation of endothelial cells to form tubes. Thus, this phenomenon is tightly controlled by positive factors such as vascular endothelial growth factor (VEGF) and negative regulators including endostatin, thrombospondin, etc., [66,67]. Neovascularization is fundamental in a variety of physiological processes such as embryonic development and pregnancy [68]. On the other hand, angiogenesis is a crucial event for tumor progression and metastatic cascade, therefore many cancer therapies are directed against the tumor-associated vasculature [69,70,71]. Recent observation showed that a series of natural compounds, including ginger extracts, were proposed as antiangiogenic/angiopreventive substances both in in vitro and in vivo [20,26]. In this contest, it was demonstrated that 6-gingerol was able to inhibit the proliferation and tube formation of human umbilical vein endothelial cells (HUVECs) in response to VEGF or bFGF [9]. Also, 6-gingerol strongly inhibited sprouting of endothelial cells in the rat aorta model and angiogenesis in the mouse cornea in response to VEGF; while in the mouse model of melanoma, i.p. administration of the above-mentioned ginger extract reduced the number of lung metastasis, with the preservation of apparently healthy behavior [9]. Kim and their collaborators showed that in phorbol ester-stimulated mouse skin, 6-gingerol was capable of inhibiting tumor promoter-induced activation of AP-1 and COX-2 expression by blocking the activation of p38 MAP kinase (p38 MAPK) and NF-κB [72]. Since p38 MAPK, NF-κB, and COX-2 are involved in angiogenesis, the anti-angiogenic activity of 6-gingerol might be due to blocking their activation.

Moreover, the use of CAM assay showed that ginger extracts were capable to reduce neovascularization as well as blood vessel diameter in a dose-dependent manner [73]. The importance of 6-gingerol in angioprevention and cancer treatment was also supported by further experimental evidence that demonstrated that the ginger extract was a potent inhibitor of endothelial cell proliferation as tube-like formation in vitro and in vivo, directly inhibiting the growth of rat YYT colon cancer cells or mouse MS1 endothelial cells in response to the growth factors derived from another colon cancer cell line (mouse CT26) [74]. Interestingly, there is an inverse dose-dependent relationship between proliferation and concentration of the ginger extract used [74].

NF-κB, as well as IL-8, plays an important role in tumorigenesis, given its ability to control the expression and function of numerous genes involved in cell proliferation, sustained angiogenesis, and evasion from apoptosis. Different tumor types, including ovarian cancer, have been shown to express high constitutive NF-κB activity [75]. It was shown that 6-gingerol treatment of cultured ovarian cancer cells induced serious growth suppression by inhibiting NF-kB activation and decreasing the VEGF and IL-8 secretion [10].

In summary, ginger derivatives seem to be potent anti-angiogenic substances that point to a possible role in preventing cancer from becoming malignant, presumably by selective inhibition of neovessel formation in tumor sites.

## 6. Cancer Stem Cells, Epithelial-Mesenchymal Transition and Ginger

In many tumors, a subpopulation of cells named cancer stem cells (CSCs) is involved in dissemination through their stemness properties. In fact, CSCs play a critical role in metastatic potential, resistance to chemotherapies, as well as the relapse of malignancies [76,77,78,79]. These cells are frequently identified in various tumors, including brain, pancreas, liver, ovary, colon, lung, skin, and prostate cancers [77,80,81]. Together with CSCs, the epithelial–mesenchymal transition (EMT) is responsible for the metastatic propensity of cancer; in fact, it is reported that EMT cells show stem cell-like facets [82,83]. The Wnt/β-catenin signaling pathway is considered to be a critical inducer of the EMT process and is important in maintaining cancer stem cell properties [84], and β-catenin is the main mediator for the Wnt signaling from the cytoplasm through the nucleus [27,85,86]. Different studies show that defective functions in the Wnt/β-catenin pathway are the key oncogene stimulus in 90% of patients affected by colon cancer and, coincidently, this pathway has a principal role in CSCs maintenance in CRC patients [76,87].

MicroRNAs (miRs) are endogenously small, noncoding RNAs that can post-transcriptionally regulate gene expression and seem to play an important role in maintaining normal cellular functions [88]. Studies have shown that the miR-200 family plays a significant role in the inhibition of the proliferation and metastases potential of CSCs and EMT phenomenon by suppressing Wnt/catenin signaling [89]. One recent study showed that zerumbone could reverse EMT to the mesenchymal–epithelial transition (MET) through the upregulation of miR-200c by decreasing β-catenin expression in CRC HCT-116 and SW-48 cell lines and by inhibiting the transcription of genes involved in EMT and CSCs [90]. As a result, CRC HCT-116 and SW-48 cell lines showed reduced cell viability after zerumbone treatment.

Another study demonstrated that ZD 2-1, a mixture of ZD 2, novel zingerone derivative, and zingerone, significantly inhibited the TGF-β1 induced an increase in migration and invasion in SNU182 hepatocellular carcinoma cells when the concentration of ZD 2-1 reached 40 µM [18]. In particular, ZD 2-1 inhibited nuclear translocation of NF-kB and activation of p42/44 MAPK/AP1 signaling pathways in the TGF-β1 induced EMT, probably by inhibiting activation of MMP-2/9 and p42/44 MAPK [87].

In another experiment, 6-shogaol was found to interfere with the Notch pathway, which is known to be actively involved in the self-renewal of CSCs. The treatment of MCF-7 and MDA-MB-231 breast cancer lines, both in monolayer and 3D spheroids configuration, with 25 μM of 6-shogaol, reduced the cleavage of Notch1 in a time-dependent manner, and consequently, decreased the Notch targets (Hes1 and Cyclin D1), in this way interfering with the stem cell self-renewal pathway [13].

Additionally, the anti-tumor effects of [10]-gingerol in vivo was validated by using metastatic 4T1Br4 tumor-bearing mice [91]. Control mice over the subsequent 14 days of treatment showed weight loss, indicative of cachexia typically observed in mice with a high tumor burden. In contrast, mice from the [10]-gingerol (10 mg/kg) group gained some weight. The results indicated a significantly lower incidence of mice with brain lesions in the [10]-gingerol-treated group (1/13) compared to controls (7/13) [91]. Moreover, [10]-gingerol reduced spontaneous lung and bone metastatic burden. In addition, [10]-gingerol was well-tolerated in vivo, induced a marked increase in caspase-3 activation, and inhibited orthotopic tumor growth in a syngeneic mouse model of spontaneous breast cancer metastasis. Importantly, by using both spontaneous and experimental metastasis assays, it was evidenced that [10]-gingerol significantly inhibited metastasis of multiple organs including lung, bone, and brain.

## 7. Ginger and Multidrug Resistance

Multidrug resistance (MDR) mechanisms are associated with increased expression of the P-glycoprotein (Pgp) or increased cellular metabolism of drug detoxifying proteins, such as glutathione-S-transferase (GST), that are correlated with increased resistance to apoptosis [24,92]. Additionally, multidrug resistance-associated protein 1 (MRP1), involved in the transport of many antitumor agents, is overexpressed in many chemoresistant cancer types including gastric cancer, neuroblastoma, and prostate cancer [93,94].

Recent research found that GST and MRP1 protein expression in the docetaxel-resistant human prostate (PC3R) cancer cell line was higher than in the docetaxel-sensitive human prostate (PC3) cancer cell line [95]. The results showed that 6-gingerol, 10-gingerol, 6-shogaol, and 10-shogaol inhibited the proliferation of PC3R cells through the downregulation of MRP1 and GST-protein expression [95]. Another study showed that a combined therapy of 6-gingerol with doxorubicin (doxo) could enhance the efficacy of doxo-based regiments in the treatment of Pgp-mediated MDR tumor with no severe side effects [12]. 6-gingerol in combination with doxo produced a significant increase of doxo accumulation (up to 44%) with the concentration of ginger at 10 and 20 µM in combination with doxo 2, 4, and 8 µM within the doxo-resistant human uterus sarcoma cell line MES-SA/Dx5. Additionally, the increase in GSH production was significant (up to 13%) at a higher (20 µM) 6-gingerol concentration [12]. So, on one hand, 6-gingerol could act as chemosensitizer inhibiting Pgp activity and, on the other hand, at high concentrations, it could have an anti-oxidative capacity that could be useful to protect MDR-negative normal cells against the damage caused by the generation of free radicals during anticancer treatment while its extrusion from resistant cells via Pgp could reduce the protective effects of cells increasing doxo sensitivity. Combined treatment of ginger oil with Methotrexate (MTX) increased the cytotoxic effect of MTX by 1.54-fold for the CCRF-CEM, T-cell Acute Lymphoblastic Leukemia (T-ALL) malignant cell line and 2.3-fold for Nalm-6 (B-ALL) cells, while the cytotoxic activity of this herbal extract in normal mononuclear cells was negligible [11]. Additionally, 11 out of 12 patient samples showed 1.2–16.5% increased apoptosis compared with the untreated samples. It was also shown that the more resistant cells were to the chemotherapy drug, the more sensitive they were to the medicinal herb. These data introduced ginger as a promising candidate for improved combination therapies in ALL, especially for those patients who show resistance to chemotherapy [11].

## 8. Ginger Enhanced Bioavailability and Combined Treatment

Low bioavailability alongside the poor solubility of ginger derivatives hinders their clinical application, probably due to poor absorption, hydrophobicity, extreme instability, and rapid metabolism, with concomitant elimination [96,97]. Recently, nanotechnologies (polymer nanoparticles or micelles, liposomes, inorganic nanoparticles, and nano-emulsions) have shown a huge advantage in enhancing bioavailability of these compounds as well as their oral absorption, reducing medicinal herb doses and toxicity, thereby improving the target ability and therapeutic effects [98,99]. Comparatively, self-assembled micelles could provide several advantages to drug delivery systems because of their high drug loading capacity, low dose of formulation required, and long circulation time [100,101]. In the recent study, the polyethylene glycol (PEG) derivative of linoleic acid (mPEG2K-LA) was first employed as a material for forming micelles to encapsulate 6-shogaol and enhance its solubility [102]. The formulated 6-shogaol loaded micelles (SMs) significantly slowed the drug release in stimulated media of the gastrointestinal tract and increased the sensitivity of tumor cells to the prototype drug. A high drug encapsulation of 80% was achieved under a drug loading capacity of 7%, which greatly enhanced the 6-shogaol delivery efficiency versus general oral delivery systems. Therefore, SMs show a slower release rate than the free 6-shogaol [102]. More importantly, the in vitro cytotoxic effect of SMs in HepG2 cells is significantly higher than free 6-shogaol [102]. In addition, SMs showed enhanced oral bioavailability and liver and brain distribution compared to free 6-shogaol. The in vivo liver protection study in mice also demonstrated that SMs markedly reduced the activities of serum AST, ALT, and liver MDA levels, while they remarkably increased the antioxidant activities (GSH-Px, T-SOD). Therefore, the novel SMs are expected to serve as a promising carrier for 6-shogaol to enhance its cancer treatment and hepatoprotection [102].

Because radiotherapy is one of the main treatment options in head and neck cancer, 6-shogaol was combined with irradiation to evaluate a possible radiosensitizing effect [16]. The results of this study showed that 6-shogaol enhanced the effect of irradiation on SCC25, CAL27, two squamous cell carcinoma cell lines of the tongue and FaDu, and a squamous cell carcinoma cell line of the pharynx in vitro. Cell viability assays showed that irradiation in combination with 6-shogaol lead to a stronger growth inhibition than each treatment method alone. Experiments demonstrated a synergistic effect of 6-shogaol and irradiation, therefore a radiosensitizing capability of 6-shogaol can be supposed [16]. A recent study described a nanovector made from ginger-derived lipids that can serve as a delivery platform for the therapeutic agent doxorubicin (doxo) to treat colon cancer [28]. The nanoparticles from ginger were created and their lipids were reassembled into ginger-derived nanovectors (GDNVs). A subsequent characterization showed that GDNVs are efficiently taken up by colon cancer cells. Modified GDNVs conjugated with the targeting ligand folic acid-mediated targeted delivery of doxo to Colon-26 tumors in a xenograft tumor model in vivo and enhanced the chemotherapeutic inhibition of tumor growth compared with free drug [28]. Such delivery vehicles have enhanced the permeability and retention effect that allowed drugs to reach tumors more passively through leaky vasculatures surrounding the mass. The result of this study demonstrated that GDNVs loaded with doxo successfully inhibited tumor growth in a Colon-26 xenograft tumor model [28].

Additionally, the studies reported the effects of combined treatment (ginger and gelam honey), which downregulate the gene expressions of Akt, mTOR, Raptor, Rictor, β-catenin, Gsk3β, Tcf4, and cyclin D1 while cytochrome C and caspase 3 genes were shown to be upregulated in HT29 colon cancer cells [103]. Furthermore, an extract mixture of turmeric, ginger, and garlic induced apoptosis in MCF-7 and ZR-75 breast cancer cell lines [104]. A combined treatment with NE mix-tamoxifen caused the extension of apoptosis indicating a potential role of the NE mix in sensitizing the ER-positive breast cancer cells towards tamoxifen. Moreover, a combined treatment with NE mix-tamoxifen altered the expression of apoptotic markers (p53 and Caspase 9) leading to apoptosis in breast cancer cell lines.

So, nanotechnologies and combined treatment seem to increase the efficiency of ginger derivatives therapeutic effects by increasing their bioavailability.

## 9. Conclusions and Future Perspectives

Nowadays, available drugs for treating cancer are often toxic, expensive, and little effective. Ginger derivatives possess high potential chemopreventive properties such as cell cycle arrest, increased cellular death (apoptosis, autophagy and autosis), as well as redox homeostasis unbalance. Furthermore, they inhibit angiogenesis, CSCs formation, and the EMT process. Therefore, this natural compound directly and indirectly influences tumor cell survival and inhibits invasion and metastasis processes, without significant toxic effects on normal cells [18,26]. Additionally, ginger enhances the therapeutic efficacy of the currently available anti-cancer drugs and represents a good adjuvant to certain phytochemical compounds including turmeric, garlic, and gelam honey [103,104]. A very important moment in ginger derivatives’ chemopreventive properties is that they do not cause side effects, but on the contrary, they ease the side effects provoked by other cancer treatments, like radio- and chemotherapy, giving ginger a considerable advantage in being considered a chemopreventive natural compound [105,106]. However, most of the known activities of ginger components have been studied only in in vitro and in vivo studies, except for a few clinical studies in human subjects. Therefore, more substantial and well-controlled clinical human studies are needed to illustrate its efficacy as an anticancer agent, since it is a safe and encouraging alternative. The recent development of nanotechnologies (polymer nanoparticles or micelles, liposomes, inorganic nanoparticles, and nano-emulsions) provides a chance to improve oral absorption, bioavailability of ginger, while also improving the target ability and its therapeutic effects [28,102]. In summary, ginger derivatives have various health effects and therapeutic properties; nevertheless, their biological applications are limited due to their hydrophobic nature. The low aqueous solubility of this compound seems to be the major obstacle for its lab-to-clinic development as a drug; therefore, it appears to be necessary to use advanced extraction methods to improve its bioavailability.

## Figures and Tables

**Table 1:** Chemopreventive activities of phenolic compounds of ginger.

Phenolic Ginger Compounds	Chemopreventive Activities	References
6-gingerol	Blockage of the cell cycle at G2/M phase; decrease of cells in the SubG0 phase; depolarization and potential subsequent deterioration of the mitochondrial membrane; induction of apoptosis; inhibition of angiogenesis; induction of growth suppression; enhancement the doxorubicin efficacy	[8,9,10,11,12]
6-paradol	Reduce blood glucose	[7]
6-shogaol	Arrest of the cell cycle in G2/M phase; decrease levels of STAT3 and NF-κB-regulated target genes including cyclin D1; induce apoptosis; downregulation of surviving; decrease tumor volume and tumor burden; restore wild type p53 function; provoke autophagy; inhibit phase I enzymes (Cyt-p450 and Cyt-b5); increase phase II enzymes (GST, GR, and GSH); reduce the cleavage of Notch1	[13,14,15,16,17]
Zingerone	Inhibition of TGF-β1 induced epithelial-mesenchymal transition, migration, and invasion	[18]

**Table 2:** Effects of ginger in different types of cancer.

Tumor Entity	Functions of Ginger	References
Breast cancer	Blockage of the cell cycle at G2/M phase; Induction of typical apoptotic changes in nuclear morphology, chromatin condensation and fragmentation, membrane shrinkage and blebbing; enabled autophagy followed by caspase-independent apoptosis; induction of autophagy	[16,18,22]
Prostate cancer	Arrest of cell cycle in the G1 phase with subsequent decrease in S and G2/M through p21 dependent pathway; downregulation of MRP1 and GST-protein expression	[23,24]
Ovarian cancer	Suppressed production of NF-κB regulated angiogenic factors; p53 stimulation of apoptosis through Bcl-2 elimination	[25,26]
Colon cancer	Arrest of cell cycle at different check points by inhibition of cyclin dependent kinases and activation of cell cycle check points; upregulation of p21 expression; reverse of EMT to Mesenchymal–epithelial transition (MET) through the upregulation of miR-200c	[17,19,27,28]
Hepatocellular carcinoma	Arrest of cell cycle at the G2/M phase; inhibition of the PI3K/AKT/mTOR and STAT3 signaling pathways; inhibition of Bcl-2 expression and up-regulation of Bax, cytochrome c, caspase-9 and -3 protein expressions	[21,29]
Gastric adenocarcinoma	Interruption of cell cycle at different check points; mediation of mitochondrial pathway of apoptosis; unbalance ROS homeostasis and induction of apoptosis	[30]
Non-small lung epithelium cancer	The loss of mitochondrial membrane potential of that leads to increase in Bax/Bcl-2 ratio and activation of mitochondrial death cascade	[31,32]
Melanoma	Induction of caspase independent cell death via the inhibition of ERK1/2, p38 and Akt signaling pathway	[33]
Endometrial adenocarcinoma	Induction of apoptosis by increasing the expression of p53 and Bax and simultaneously decreasing the expression of Bcl-2	[34]
Cervical cancer	Induction of typical apoptotic changes in nuclear morphology, chromatin condensation and fragmentation, membrane shrinkage and blebbing	[35]
Lung cancer	Sensitization of TRAIL-induced apoptosis by inhibiting autophagy flux	[36]
Head and neck squamous carcinoma	Increase in apoptotic death by downregulation of surviving; inhibition of mutant p53 Bcl-2 expression, and increased expression of Bax, regulation of Bax/Bcl-2 ratio which induce cell apoptosis	[37,38]
Pancreatic cancer	Activation of AMPK, a positive regulator of autophagy, and inhibition mTOR, a negative autophagic regulator; unbalance ROS homeostasis and induction of autosis	[39]

Molecular action of ginger and its derivatives in different kinds of cancer.

## Abbreviations

ALT	alanine aminotransferase
AP-1	activator protein 1
AST	aspartate aminotransferase
ATPase	adenosine triphosphatase
B-ALL	B-cell Acute Lymphoblastic Leukemia
Bcl-2	B-cell lymphoma 2
CAM	chorioallantoic membrane
Caspases	cysteine-aspartate proteases
CAT	catalase
CDK	cyclin-dependent kinases
CFEZO	crude flavonoid extracts from Z. officinale
CKI	cyclin-dependent kinases inhibitor
COX-2	cyclooxygenase-2
CRC	colorectal carcinoma
CSCs	cancer stem cells
CuZn-SOD Cu-Zn	superoxide dismutase
CYP-P450	cytochrome P450
DMBA	0.5% 7,12-dimethylbenz[a]anthracene
DNA	deoxyribonucleic acid
DNPH	dinitrophenylhydrazone
ECOG	Eastern Cooperative Oncology Group
EE	Etlingera elatior
EMT	epithelial-mesenchymal transition
eNO	endothelial nitric oxide synthase
ERK	Extracellular signal-regulated kinase
FAs	focal adhesions
GAE	aqueous extract of ginger
GDNVs	ginger-derived nanovectors
GR	glutathione reductase
GSH	Glutathione
GST	glutathione-S-transferase
HBP	hamster buccal pouch
HNSCC	head and neck squamous cell carcinoma
HUVECs	human umbilical vein endothelial cells
IAP	inhibitor of apoptosis
IL-8	interleukin 8
LC3-II	light chain3-II
MAPK	Mitogen-Activated Protein Kinase
MDA	malondialdehyde
MDR	multidrug resistance
MET	esenchymal–epithelial transition
miRs	microRNA
MMP	Matrix metalloproteinase
MRP1	multidrug resistance-associated protein 1
mTOR	the mechanistic target of rapamycin
MTX	methotrexate
NADPH	Nicotinamide adenine dinucleotide phosphate
NF-κB	nuclear factor kappa-light-chain-enhancer of activated B cells
Nox	NADPH oxidases
NSCLC	non-small-cell lung cancer
PEG	Polyethylene Glycol
Pgp	P-glycoprotein
PI3K	phosphatidylinositol-3-kinase
RNA	ribonucleic acid
ROS	reactive oxygen species
SCID	severe combined immune deficient
SDGE	Steam Distilled Extract of Ginger
SMs	6-shogaol loaded micelles
SSHE	extract of Syussai ginger
STAT3	signal transducer and activator of transcription 3
T-ALL	T-cell Acute Lymphoblastic Leukemia
TNBC	triple negative breast cancer
TNF	tumor necrosis factor
TRAIL	TNF-related apoptosis-inducing ligand
USP14	ubiquitin-specific peptidase 14
VEGF	vascular endothelial growth factor
XO	xanthine oxidase
ZOME	Methanolic extract of Zingiber officinale rhizome
